# Predictors of contrast-induced acute kidney injury in patients with coronary artery disease receiving contrast agents twice within 30 days

**DOI:** 10.1186/s40779-020-00243-x

**Published:** 2020-03-26

**Authors:** Chong-Huai Gu, Xiao-Zeng Wang, Ya-Ling Han, Quan-Min Jing, Li-Li Ren, Yan Zhang, Jun-Yin Peng, Xin Zhao

**Affiliations:** 1 Cardiovascular Research Institute and Department of Cardiology, General Hospital of Shenyang Military Region, Shenyang, 110840 China; 2 Department of Cardiovascular, Anqing Municipal Hospital, Anqing, 246000 Anhui China

**Keywords:** Predictors, Contrast-induced acute kidney injury, Coronary artery disease

## Abstract

**Background:**

None of study mentioned about contrast-induced acute kidney injury (CI-AKI) in people who have received contrast agents twice within in a short period of time. This study is trying to identify the predictors.

**Methods:**

We enrolled 607 patients between Oct. 2010 and Jul. 2015 who received contrast agents twice within 30 days in the Department of Cardiology of the General Hospital of Shenyang Military Region. The primary outcome was CI-AKI within 72 h after contrast agent exposure. Patients were divided into groups A (*n* = 559) and group B (*n* = 48) according to whether CI-AKI occurred after the second agent.

**Results:**

Patients in group B (CI-AKI occurred after the second agent) had a more rapid heart rate and more usage of diuretics and digitalis. In group B, CI-AKI occurred more frequently after the first agent. Multivariate logistic regression showed that diuretic (*P =* 0.006) and intra-aortic balloon pump (IABP) usage (*P =* 0.012) were independent predictors of CI-AKI after the first agent. Angiotensin-converting enzyme inhibitor/Angiotensin II receptor antagonist (ACEI/ARB) usage (*P =* 0.039), IABP usage (*P =* 0.040) and CI-AKI occurring after administration of the first agent (*P =* 0.015) were independent predictors of CI-AKI after the second. Furthermore, dividing the patients into tertiles of the time interval between the two agents showed that CI-AKI occurred more frequently when the second agent was administered within 1–3 days after the first exposure than within 4–6 days (12.4% vs. 5.0%, *P =* 0.008) or ≥ 7 days (12.4% vs. 6.4%, *P =* 0.039).

**Conclusions:**

Diuretic and IABP usage are independent predictors of CI-AKI following exposure to a first contrast agent. The major predictors of CI-AKI after exposure to a second agent are time since the first contrast exposure, ACEI/ARB usage, and IABP usage. More importantly, a three-day interval between the two agents is associated with a higher incidence of CI-AKI following the second administration.

## Background

Contrast-induced acute kidney injury (CI-AKI) is a familiar complication experienced by patients with coronary artery disease (CAD) who are administered iodine-containing contrast agents for procedures such as coronary angiography (CAG) and percutaneous coronary intervention (PCI) [1–3].

CI-AKI has been proven to be associated with risk indices of longer hospitalization and short- and/or long-term mortality [2, 4, 5]. The incidence of CI-AKI is approximately 0.6–2.3% [6]. However, this value varies depending on the presence of risk factors, and several clinical predictors of CI-AKI have been introduced [7, 8]. Additionally, CI-AKI has been linked with a worse clinical outcome in patients with acute coronary syndrome (ACS) [5, 8–10]. Well-established risk factors of CI-AKI include diabetes, congestive heart failure, acute hypotension, advanced age, ST-elevation myocardial infarction (STEMI), volume depletion, the amount of contrast administered, the type of contrast media, and the simultaneous use of nephrotoxic medications in unselected patients after CAG or PCI [7, 11, 12]. However, those studies mainly focused on the effect of a one-time exposure to a contrast agent on in-hospital or long-term endpoints [5, 8, 9].

It is becoming more common for patients with CAD to receive a contrast agent two or more times (CAG or PCI) because of delay-PCI, rescue-PCI or other reasons. The aim of our investigation was to evaluate predictors of the development of CI-AKI in patients who received contrast agents twice within 30 days and the association between CI-AKI and adverse clinical outcomes.

## Methods

### Study population

This was a single-center, retrospective, observational study. All research adhered to the tenets of the Declaration of Helsinki, and compliance was maintained throughout the study. The subjects were recruited from the cardiovascular catheterization database of the General Hospital of Shenyang Military Region between Oct. 2010 and Jul. 2015. The total number of patients who underwent CAG or PCI was 23,444. Of these patients, 607 received contrast agents twice within a 30-day period. All patients provided written informed consent. All experimental and operational procedures were performed using standard interventional techniques. Cardiogenic shock (CS) patients were excluded. CS is defined below. Patients were divided into two groups according to whether CI-AKI occurred following exposure to the second agent: group A (*n* = 559), no occurrence of CI-AKI following the second agent, and group B (*n* = 48), occurrence of CI-AKI following the second agent.

### Definitions

The primary outcome was CI-AKI after any of the contrast agents. CI-AKI was diagnosed based on the Risk, Injury, Failure, Loss of kidney function and End-stage kidney disease/Acute Kidney Injury Network (RIFLE/AKIN) criteria, which were defined as a serum creatinine level increase of 25% or creatinine 0.5 mg/dl or more above baseline within 72 h after the administration of the contrast agent [13, 14]. The estimated glomerular filtration rate (eGFR) was calculated using the equation from the Modification of Diet in Renal Disease formula: eGFR =175 × serum creatinine^-1.154^× age^-0.203^× (0.742, if female) [15]. Chronic kidney disease (CKD) was defined as an eGFR< 60 ml/(min·1.73 m^2^) for at least 3 months [16]. Serum creatinine was measured before the procedure and daily for 3 days after the administration of the contrast agent. ACS was defined according to the guidelines of the European Society of Cardiology (ESC) [17]. Patients at high risk of CI-AKI after exposure to any of the contrast agents routinely received intravenous hydration treatment with saline solution administered at 1 ml/(kg·h) for 12 h after exposure to the contrast agent. Anemia was defined as hemoglobin (Hb) of less than 12 g/dl in females and 13 g/dl in males according to the World Health Organization’s recommendation [18]. Positive cardiac markers at admission were defined as an elevated serum troponin I (cut-off of 0.15 ng/ml) and/or a creatine kinase-myocardial band (CK-MB) (cut-off of 24 U/L). ST elevation on the admission electrocardiogram (ECG) was defined as recommended by the ESC guidelines [19]. CS was defined as a systolic blood pressure of less than 85 mmHg with evidence of decreased organ perfusion caused by severe left ventricular dysfunction, right ventricular infarction or mechanical complications of the infarction [9].

### Statistical analysis

Continuous variables are presented as the mean ± standard deviation (SD) or the median, as appropriate, and categorical variables are given as percentages (%). The *t*-test or Mann-Whitney *U* test was used to compare continuous variables between two groups, and one-way ANOVA was used to compare categorical variables between groups. The chi-squared test was used to compare the rates of outcomes. Basic data were analyzed by using IBM SPSS version 20.0. Differences in demographic variables were evaluated using the chi-squared test. Non-normally distributed continuous variables, presented as medians and interquartile ranges, were analyzed using the Wilcoxon rank-sum test. Logistic regression analysis was used to calculate odds ratios for the comparison of CI-AKI rates between groups. Multivariate analysis for CI-AKI included the results with values of *P <* 0.05 in the univariate analysis or those that could potentially affect the clinical outcome according to our experience. Survival analysis was used to compare patients who developed CI-AKI and those who did not develop CI-AKI following administration of the second agent using the Kaplan-Meier estimator. All *P* values were two tailed, and statistical significance was defined by a *P* value < 0.05.

## Results

The 607 consecutive patients exposed to contrast agents twice within 30 days and who had complete clinical data were separated into two groups (A and B) according to whether CI-AKI occurred following the second exposure. Forty-eight (7.9%) patients developed CI-AKI after the second administration. Baseline characteristics, laboratory results and medications for the two groups are listed in Table 1. Patients in group B exhibited a faster in-hospital heart rate (81.4 ± 17.3 beat/min vs. 76.4 ± 14.1 beat/min, *P =* 0.022). Diuretics (56.3% vs. 37.6%, *P =* 0.011) and digitalis (35.4% vs. 18.4%, *P =* 0.005) were also used more frequently in group B. The other variables did not differ significantly between the two groups.

**Table 1:** Baseline patient characteristics in two groups

Item	Group A (*n* = 559)	Group B (*n* = 48)	*P* value
Age (year, *x* ± *s*)	60.2 ± 11.0	61.5 ± 12.7	0.438
Female [*n*(%)]	121 (21.6)	14 (29.1)	0.229
Weight (kg, *x* ± *s*)	71.7 ± 11.7	72.1 ± 11.7	0.821
Height (cm, *x* ± *s*)	169.9 ± 6.6	170.3 ± 6.7	0.699
BMI (kg/m^2^, *x* ± *s*)	24.8 ± 3.6	24.9 ± 3.7	0.930
Current smoker [*n*(%)]	288 (51.5)	27 (56.2)	0.529
Current alcohol [*n*(%)]	180 (32.2)	14 (29.1)	0.665
STEMI [*n*(%)]	196 (35.0)	22 (45.8)	0.136
Hypertension [*n*(%)]	303 (54.2)	33 (68.7)	0.061
Diabetes [*n*(%)]	132 (23.6)	16 (33.3)	0.132
CKD [*n*(%)]	34 (6.1)	6 (12.5)	0.119
Stroke [*n*(%)]	53 (9.5)	8 (16.7)	0.130
Cancer [*n*(%)]	4 (0.7)	0	1.000
Peripheral vascular disease [*n*(%)]	7 (1.3)	2 (4.2)	0.109
Ulcer [*n*(%)]	58 (10.4)	4 (8.3)	0.807
NYHA class II-III [*n*(%)]	109 (19.5)	15 (31.3)	0.053
SBP (mmHg, *x* ± *s*)	133.9 ± 21.9	136.8 ± 22.4	0.379
DBP (mmHg, *x* ± *s*)	79.3 ± 13.4	80.85 ± 13.5	0.426
HR (beat/min, *x* ± *s*)	76.4 ± 14.1	81.4 ± 17.3	0.022
Basic-Scr (μmol/L, *x* ± *s*)	77.4 ± 25.0	75.1 ± 24.5	0.548
Basic-eGFR [ml/(min•1.73m^2^), *x* ± *s*]	108.4 ± 41.5	110.0 ± 43.0	0.908
BUN (mmol/L, *x* ± *s*)	5.9 ± 1.9	6.1 ± 1.3	0.638
K^+^ (mmol/L, *x* ± *s*)	4.1 ± 0.4	4.2 ± 0.3	0.243
Na^+^ (mmol/L, *x* ± *s*)	140.1 ± 3.4	139.6 ± 3.0	0.391
PLT (× 10^9^/L, *x* ± *s*)	208.8 ± 55.8	209.5 ± 55.4	0.943
WBC (×10^9^/L, *x* ± *s*)	8.3 ± 3.1	8.3 ± 2.8	0.995
Hb (g/L, *x* ± *s*)	137.9 ± 24.1	136.2 ± 17.4	0.671
TC (mmol/L, *x* ± *s*)	4.2 ± 1.3	4.1 ± 1.0	0.819
TG [mmol/L, M(Q_1_, Q_3_)]^a^	1.48 (1.13,1.72)	1.44 (0.72,1.98)	0.367
ALT [mmol/L, M(Q_1_, Q_3_)]^a^	26.00 (13,81)	24.00 (9,63)	0.975
AST [mmol/L, M(Q_1_, Q_3_)]^a^	25.00 (7,76)	23.00 (4,67)	0.250
ALB (mmol/L, *x* ± *s*)	33.2 ± 20.5	35.3 ± 15.5	0.718
HDL-C (mmol/L, *x* ± *s*)	1.2 ± 0.4	1.0 ± 0.3	0.684
LDL-C (mmol/L, *x* ± *s*)	2.4 ± 1.0	2.4 ± 0.9	0.790
Glucose (mmol/L, *x* ± *s*)	6.6 ± 2.9	7.2 ± 3.4	0.337
CK [U/L, M(Q_1_, Q_3_)]^a^	137.00 (27.31,452.50)	108.00 (45.79,168.75)	0.303
CK-MB [U/L, M(Q_1_, Q_3_)]^a^	17.00 (7.14,22.75)	17.00 (6.34,16.75)	0.936
TNT [ng/L, M(Q_1_, Q_3_)]^a^	0.05 (0.01,0.523)	0.05 (0.01,0.162)	0.241
NT-proBNP [μg/L, M(Q_1_, Q_3_)]^a^	83.00 (12.00,1085.00)	140.00 (21.00,2050.00)	0.236
LV (mm, *x* ± *s*)	48.8 ± 6.4	49.8 ± 5.8	0.687
EF (%, *x* ± *s*)	58.9 ± 10.7	57.6 ± 11.6	0.730
Medicine care [*n*(%)]			
Diuretics	210 (37.6)	27 (56.3)	0.011
CCB	231 (41.3)	21 (43.8)	0.743
β-RB	469 (83.9)	40 (83.3)	0.918
ACEI/ARB	444 (79.4)	34 (70.8)	0.162
Statins	366 (65.5)	37 (77.1)	0.102
Digitalis	103 (18.4)	17 (35.4)	0.005

*Group A* No CI-AKI from second agent, *Group B* CI-AKI from second agent, *BMI* Body mass index, *STEMI* ST-segment elevation myocardial infarction, *CKD* Chronic kidney disease, *NYHA* New York Heart Association, *SBP* Systolic blood pressure, *DBP* Diastolic blood pressure, *HR* Heart rate, *Scr* Serum creatinine, *eGFR* Estimated glomerular filtration rate, *BUN* Blood urea nitrogen, *PLT* Platelets, *WBC* White blood cells, *Hb* Hemoglobin, *TC* Total cholesterol, *TG* Triglycerides, *ALT* Alanine aminotransferase, *AST* Aspartate aminotransferase, *ALB* Albumin, *HDL-C* High-density lipoprotein cholesterol, *LDL-C* Low-density lipoprotein cholesterol, *CK* Creatine kinase, *CK-MB* Creatine kinase-myocardial band, *TNT* Troponin T, *NT-proBNP* N-terminal pro-brain natriuretic peptide, *LV* Left ventricular, *EF* Ejection fraction, *CCB* Calcium channel blocker, *β-RB* β-receptor blocker, *ACEI* Angiotensin-converting enzyme inhibitor, *ARB* Angiotensin II receptor antagonist

^a^Means nonnormally distributed continuous variables

A review of the procedural details revealed that there was no significant difference between the two groups in the operative approach or the amount of contrast media used during the procedure. In addition, the number of stent implantations and lesions matched well. Nonetheless, CI-AKI occurred more frequently after exposure to the first contrast agent in group B (31.3% vs. 7.3%, *P <* 0.001). Furthermore, the maximal serum creatinine level was higher in group B than in group A following exposure to the second agent (100.1 ± 27.3 μmol/L vs. 76.6 ± 27.7 μmol/L, *P <* 0.001, Table 2).

**Table 2:** Agent procedural characteristics in two groups

Item	Group A (*n* = 559)	Group B (*n* = 48)	*P* value
TRI [*n*(%)]			
TRI in first agent	426 (76.2)	34 (70.8)	0.404
TRI in second agent	433 (77.5)	35 (72.9)	0.472
Agents interval time [d, M(Q_1_, Q_3_)]^a^	5 (3, 8)	4 (3, 6)	0.218
CV in 1st agent [ml, M(Q_1_, Q_3_)]^a^	100.00 (40.00, 140.00)	90.00 (50.00, 75.00)	0.128
CV in 2nd agent[ml, M(Q_1_, Q_3_)]^a^	150.00 (50.00, 100.00)	150.00 (50.00, 100.00)	0.334
Number of stent (*x* ± *s*)	2.23 ± 1.58	1.82 ± 1.19	0.096
Multivessel disease [*n*(%)]	392 (70.1)	37 (77.1)	0.310
LM lesion [*n*(%)]	11 (1.9)	3 (6.3)	0.091
Basic SYNTAX score (*x* ± *s*)	17.2 ± 7.5	19.2 ± 8.4	0.082
Residual SYNTAX score (*x* ± *s*)	8.8 ± 5.8	11.1 ± 7.0	0.073
Complete revascularization [*n*(%)]	353 (63.2)	24 (50.0)	0.072
CI-AKI post-first agent [*n*(%)]	41 (7.3)	15 (31.3)	< 0.001
Serum creatinine (μmol/L, *x* ± *s*)			
Baseline	77.4 ± 25.0	75.1 ± 24.5	0.548
Maximal post-first agent	84.1 ± 33.9	93.7 ± 39.4	0.094
Pre-second agent	84.4 ± 56.6	81.3 ± 24.6	0.729
Maximal post-second agent	76.6 ± 27.7	100.1 ± 27.3	< 0.001

*Group A* No CI-AKI from second agent, *Group B* CI-AKI from second agent, *TRI* Trans-radial intervention, *CV* Contrast volume, *LM* Left main, *SYNTAX* Synergy between percutaneous coronary intervention with Taxus and cardiac surgery

^a^Means non-normally distributed continuous variables

Binary logistic regression was performed to identify predictors of CI-AKI in patients who received a contrast agent twice. In the multivariate regression model, the independent predictors for the occurrence of CI-AKI after the first contrast agent were diuretic (*P =* 0.006) and IABP usage (*P =* 0.012, Table 3), while for the second agent, CI-AKI indices from the multivariate regression model revealed that the independent predictors were the time interval between exposure to the two agents (*P =* 0.037), ACEI/ARB usage (*P =* 0.039), IABP usage (*P =* 0.040) and the occurrence of CI-AKI following the first agent (*P =* 0.015, Table 4).

**Table 3:** Regression analysis for CI-AKI predictors of the first agent

Variable	Univariate analysis	Multivariate analysis		
	*P* value	*OR*	95% CI	*P* value
Female	0.854	1.026	0.424–2.484	0.954
NYHA Class II-III	0.004	1.326	0.542–3.248	0.536
Hypertension	0.626	1.671	0.736–3.796	0.220
Diabetes	0.276	1.179	0.483–2.876	0.718
Diuretics	< 0.001	3.761	1.462–9.675	0.006
CCB	0.228	0.269	0.086–1.838	0.240
β-RB	0.715	0.399	0.157–1.011	0.053
ACEI/ARB	0.757	0.575	0.240–1.377	0.214
Statins	0.518	0.643	0.097–3.304	0.390
Digitalis	0.010	1.588	0.608–4.149	0.345
IABP	< 0.001	4.245	1.378–13.083	0.012
LM lesion	0.716	0.564	0.256–1.241	0.155
Age	0.381	1.000	0.965–1.035	0.983
Basic SYNTAX score	0.078	0.996	0.946–1.047	0.861

*NYHA* New York Heart Association, *CCB* Calcium channel blocker, *β-RB* β-receptor blocker, *ACEI* Angiotensin-converting enzyme inhibitor, *ARB* Angiotensin II receptor antagonist, *IABP* Intra-aortic balloon pump, *LM* Left main, *SYNTAX* Synergy between percutaneous coronary intervention with Taxus and cardiac surgery

**Table 4:** Regression analysis for CI-AKI predictors after second agent

Variable	Univariate analysis	Multivariate analysis		
	*P* value	*OR*	95% CI	*P* value
Interval time	0.033	1.170	1.980–2.370	0.037
Female	0.232	1.460	0.660–3.226	0.350
NYHA class II-III	0.056	1.381	0.576–3.309	0.469
Hypertension	0.064	2.107	0.929–4.774	0.074
Diabetes	0.135	1.296	0.582–2.888	0.526
Diuretics	0.012	1.268	0.489–3.288	0.625
CCB	0.743	1.329	0.601–2.939	0.482
β-RB	0.918	0.698	0.277–1.759	0.446
ACEI/ARB	0.166	0.428	0.191–0.958	0.039
Statins	0.106	4.203	0.809–21.846	0.088
Digitalis	0.006	1.291	0.453–3.677	0.632
IABP	0.001	3.302	1.055–10.335	0.040
LM lesion	0.164	0.880	0.356–2.177	0.782
Age	0.438	0.996	0.963–1.031	0.838
Basic SYNTAX score	0.083	1.006	0.952–1.063	0.838
CI-AKI post-first agent	< 0.001	3.454	1.278–9.333	0.015

*NYHA* New York Heart Association, *CCB* Calcium channel blocker, *β-RB* β-receptor blocker, *ACEI* Angiotensin-converting enzyme inhibitor, *ARB* Angiotensin II receptor antagonist, *IABP* Intra-aortic balloon pump, *LM* Left main, *SYNTAX* Synergy between percutaneous coronary intervention with Taxus and cardiac surgery, *CI-AKI* Contrast-induced acute kidney injury

**Fig. 1:**
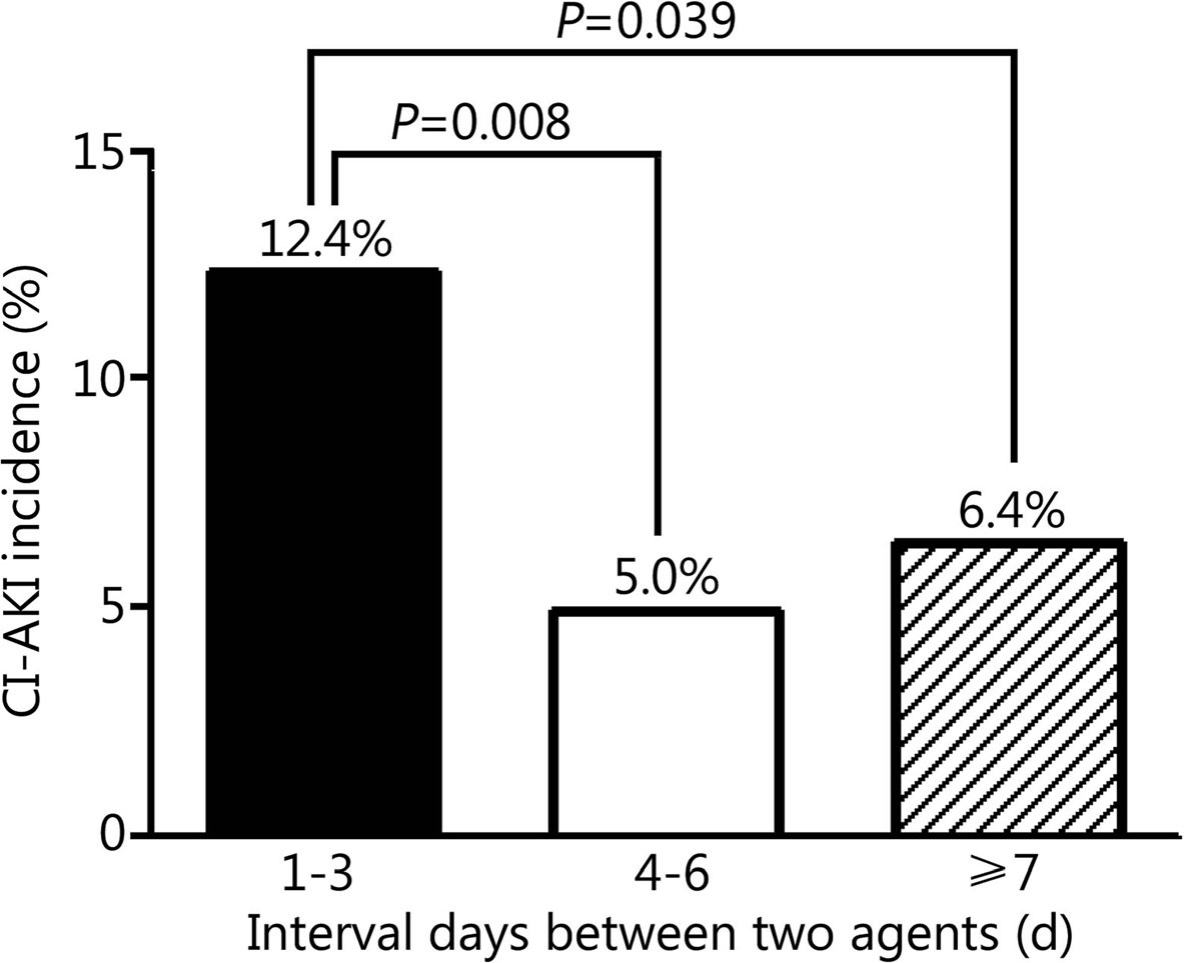
CI-AKI occurrence after the 2nd agent by tertile of the interval between the two contrast agent exposures

We separated patients by tertiles of the interval between the two agents: 1–3 days, 4–6 days and ≥ 7 days between exposures. The incidence of CI-AKI following the second agent was significantly higher in the 1–3-day group than 4–6-day group (12.4% vs. 5.0%, *P =* 0.008) and ≥ 7 day group (12.4% vs. 6.4%, *P =* 0.039, Fig. 1).

**Fig. 2:**
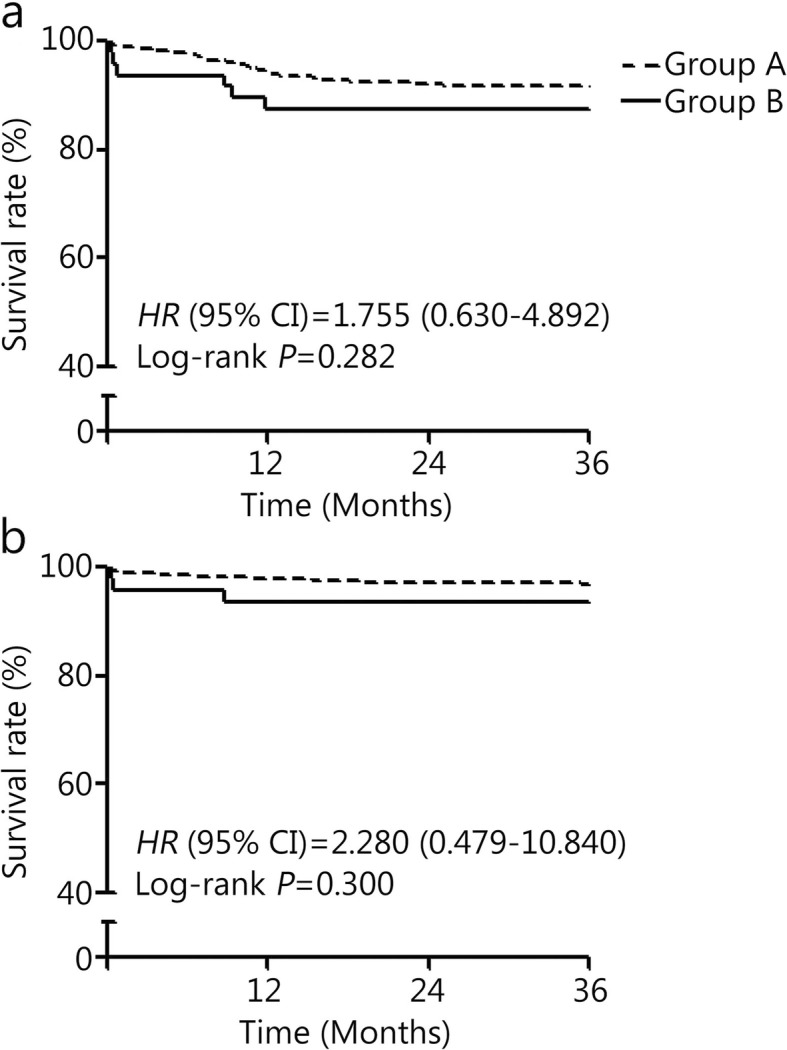
Free survival analysis from MACE (**a**) and all-cause death (**b**)

All patients had complete clinical follow-up data. The median follow-up duration was 37 months (interquartile range: 23 to 61). The incidence of all-cause death was similar between the two groups (3.8% vs. 4.2%, *P =* 0.426). Total major adverse cardiovascular events (MACE) appeared in 51 (9.1%) group A patients and 6 (12.5%) group B patients (*P =* 0.438). Among these participants, cardiac death occurred in 22 patients (group A vs. group B = 3.4% vs. 4.2%, *P =* 0.154) during the follow-up period. Only 3 patients (group A) had a myocardial infarction during the follow-up period. The incidence of in-stent restenosis was also not significantly different between the two groups (5.0% vs. 6.3%, *P =* 0.728, Table 5). After adjusting for clinically and statistically relevant covariates, the incidence of MACE and all-cause death was higher in group B, although this difference was not significant (Fig. 2).

**Table 5:** Clinical follow-up after contrast medium administration [*n*(%)]

Variable	Group A (*n* = 559)	Group B (*n* = 48)	*P* value
All cause death	21 (3.8)	3 (4.2)	0.426
MACE	51 (9.1)	6 (12.5)	0.438
Cardiac death	19 (3.4)	3 (4.2)	0.154
Myocardial infarction	3 (0.5)	0	1.000
In-stent restenosis	28 (5.0)	3 (6.3)	0.728
Stent thrombosis	1 (0.2)	0	1.000

*Group A* No CI-AKI from 2nd agent, *Group B* CI-AKI from 2nd agent, *MACE* Major Adverse Cardiovascular Events

## Discussion

In the present study, we aimed to investigate the possible predictors of CI-AKI in patients who received a contrast agent twice within 30 days. The major findings of this study are as follows: 1) diuretic (*P =* 0.006) and IABP usage (*P =* 0.012) were strongly associated with the development of CI-AKI following administration of the first contrast agent; 2) the time interval between the two procedures (*P =* 0.037), ACEI/ARB usage (*P =* 0.039), IABP usage (*P =* 0.040) and the occurrence of CI-AKI after the first procedure (*P =* 0.015) were independent predictors of CI-AKI following exposure to the second contrast agent; and 3) if the time interval between the two procedures was 3 days or less, then CI-AKI following the second contrast agent was more likely.

The main finding of the present study is the identification of several independent risk factors for CI-AKI in patients receiving a contrast agent twice within a short period of time. Some of the established risk factors have also been corroborated in our study, such as diuretics and IABP usage [20, 21]. We found that IABP usage could also increase the incidence of CI-AKI after the second contrast agent. The reason for this finding may be similar to those reported in previous literature. Those findings mainly relate to the nature of intra-arterial injection, the high volume of the contrast, the patients’ advanced age, and the severity of the patients’ illness, such as a greater number of comorbid conditions, more advanced vascular disease, hypertension, and diabetes [22, 23].

The relationship between ACEI/ARB and CI-AKI is still controversial. Some investigators have pointed out that the use of ACEI/ARB may protect the kidneys against the effects of CI-AKI [24, 25]. Recently, Duan et al. [26] proposed that ACEIs can prevent CI-AKI. Nonetheless, opposing results have been published over the past few years. For example, Kiski et al. [27] found that patients taking ACEIs/ARBs developed CI-AKI significantly more often within 72 h after contrast media administration. This observation is consistent with our results, which showed that the use of ACEIs/ARBs was associated with an increased incidence of CI-AKI following exposure to the second contrast agent.

We also found that if patients developed CI-AKI following the first procedure, then they were more likely to develop CI-AKI after the second procedure. It is well known that the mechanism of CI-AKI is mainly caused by the toxic properties of the contrast media, such as osmolality, viscosity, and ionic strength. The cytotoxicity of contrast agents is probably caused by iodine and leads to apoptosis and cell death of both endothelial and tubular cells [28]. Therefore, in patients who have recently developed one episode of CI-AKI, contrast toxicity and other factors may affect kidney function, increasing the likelihood of more frequent episodes of CI-AKI following the second exposure.

In the present study, we newly identified that the time interval between two exposures to contrast agents is an independent predictor of CI-AKI following the second procedure. Further analysis showed that the incidence of CI-AKI during the second perioperative period was significantly higher if the second exposure occurred within 3 days rather than 4–6 days or ≥ 7 days. In the 2015 ESC congress, Park et al. [29] reported a similar result when evaluating the safe time interval between exposure to multidetector computed tomography (MDCT) and coronary revascularization regarding the risk of CI-AKI. Their study pointed out that CI-AKI was more frequent in the earlier time interval in the PCI group. Furthermore, multivariate analysis identified a short interval between MDCT and PCI as a unique independent predictor of CI-AKI (≤2 days vs. > 14 days: *HR* = 2.37, 95% CI: 1.105–5.098, *P =* 0.018; 3–14 days vs. > 14 days: *HR* = 2.07, 95% CI: 0.960–4.445, *P =* 0.064). Some articles have reported that serum creatinine will usually peak 2–3 days following injection of a contrast agent and then slowly return to baseline within 14 days [30, 31]. Our result may have been because a repeat injection of contrast agent within 3 days (72 h) of the first exposure occurred at the time of peak renal toxicity caused by the previous agent. Therefore, such a strategy of repeat procedures may expose kidneys to a higher risk of CI-AKI.

Other factors, such as diabetes and left main coronary artery lesions, have been reported to be strong risk factors for CI-AKI in previous studies [2, 32]. Though the results of our multivariate analysis did not find similar correlations, trends were evident for several of these factors. In addition, the lack of significant associations may be related to the small sample size of group B.

Recent studies have demonstrated that for patients who develop CI-AKI following exposure to a contrast agent, the in-hospital and long-term adverse clinical outcomes can be attributed to cardiovascular instability and the resulting complications [2, 5, 33]. In our study, the clinical adverse events were more numerous for patients who developed CI-AKI after the second contrast agent. These findings seem to be consistent with those of previous studies [34, 35]. However, patients who developed CI-AKI after the second contrast agent did not have a significantly worse clinical prognosis during the follow-up period. This finding might be explained by the fact that some patients in group A developed CI-AKI after the first agent, while some in group B did not, which could have affected the results. The small sample size of patients receiving contrast media twice may also have impacted death or MACE incidence in this study.

Our study has several limitations. First, this study used a nonrandomized, retrospective, single-center design, which may have significantly affected the results due to confounding factors. Second, we excluded some patients whose information regarding contrast media usage and serum creatinine levels during the procedures was lost, which could potentially result in serious selection bias. Additionally, atrial fibrillation has been notoriously recognized as a predictor of poor prognostic outcomes, but we have not analyzed arrythmia types such as atrial fibrillation or any others. We have added this to the limitation section, and will analyze arrythmia in future papers [36]. Finally, in the receiver operator characteristic curve analysis, our cut-off value for predicting the development of CI-AKI in patients who received a contrast agent twice within 30 days had a slightly low sensitivity. This may be caused by the small sample size. Nevertheless, our multivariate analysis showed that the time interval between administration of the two contrast agents was one of the major predictors for developing CI-AKI, even after adjusting for various confounding factors. Further multicenter, large-scale and randomized studies may be needed to confirm this observation.

## Conclusions

In conclusion, there are several major predictors of CI-AKI in patients who receive a contrast agent twice within 30 days. Diuretic and IABP usage are independent predictors of CI-AKI following exposure to the first contrast agent. The major predictors of CI-AKI after the second contrast agent are the time interval between exposures, ACEI/ARB usage, and IABP usage. Furthermore, if the interval between two procedures is 3 days or less, then CI-AKI following the second administration of a contrast agent is more likely.

## Data Availability

The datasets used and/or analyzed during the current study are not publicly available. Access from the corresponding author’s institution can be requested by completion and approval of a Data Sharing Agreement Application.

## Abbreviations

ACS: Acute coronary syndrome
ACEI: Angiotensin-converting enzyme inhibitor
ALB: Albumin
ALT: Alanine aminotransferase
ARB: Angiotensin II receptor antagonist
AST: Aspartate aminotransferase
BMI: Body mass index
β-RB: β-receptor blocker
BUN: Blood urea nitrogen
CAD: Coronary artery disease
CAG: Coronary angiography
CCB: Calcium channel blocker
CI-AKI: Contrast-induced acute kidney injury
CK: Creatine kinase
CKD: Chronic kidney disease
CK-MB: Creatine kinase-myocardial band
CS: Cardiogenic shock
CV: Contrast volume
DBP: Diastolic blood pressure
ECG: Electrocardiogram
EF: Ejection fraction
eGFR: Estimated glomerular filtration rate
Hb: Hemoglobin
HDL-C: High-density lipoprotein cholesterol
HR: Heart rate
IABP: Intra-aortic balloon pump
LDL-C: Low-density lipoprotein cholesterol
LM: Left main
LV: Left ventricular
MACE: Major adverse cardiovascular events
MDCT: Multidetector computed tomography
NT-proBNP: N-terminal pro-brain natriuretic peptide
NYHA: New York Heart Association
PCI: Percutaneous coronary intervention
PLT: Platelets
SBP: Systolic blood pressure
Scr: Serum creatinine
STEMI: ST-segment elevation myocardial infarction
SYNTAX: Synergy between percutaneous coronary intervention with Taxus and cardiac surgery
TC: Total cholesterol
TG: Triglycerides
TNT: Troponin T
TRI: Trans-radial intervention
WBC: White blood cells
